# Role of the Gut Microbiome in Vertebrate Evolution

**DOI:** 10.1128/mSystems.00174-17

**Published:** 2018-03-20

**Authors:** Thomas J. Sharpton

**Affiliations:** a Department of Microbiology, Oregon State University, Corvallis, Oregon, USA; b Department of Statistics, Oregon State University, Corvallis, Oregon, USA

**Keywords:** coevolution, evolution, metabolome, metagenome, microbiome, vertebrates

## Abstract

Darwin referred to life as a struggle. Organisms compete for limited resources in nature, and their traits influence the outcome. Victory carries great weight as winners survive, reproduce, and progenate subsequent generations. Consequently, organismal traits that influence fitness drive adaptation and their discovery clarifies evolution. Recent research implicates the vertebrate gut microbiome as an agent of fitness, selection, and evolution. Going forward, we must define the functional effects of the gut microbiome to determine how it impacts evolution. Specifically, we must quantify how gut microbiome function diversifies in concert with vertebrate radiation and resolve specific functions that influence natural selection. In so doing, we can discover and potentially capitalize upon the mechanisms by which our gut microbiomes impact our physiology and fitness. Ultimately, we may come to find that while life involves struggle, it also depends upon cooperation.

## Abstract (precis)

Darwin referred to life as a struggle. Organisms compete for limited resources in nature, and their traits influence the outcome.

## PERSPECTIVE

Nature selects for individuals based upon the relative fitness of their phenotypes. Accordingly, evolutionary biology has focused on discovering the genomic determinants of fitness, with the goal of uncovering loci that drive adaptation. Recent research demonstrates that the diverse and abundant community of microorganisms that reside within the vertebrate gut executes a variety of functions that impact phenotype; these microbes influence nutrition, detoxify xenobiotics, stimulate gut and immune development, and modulate behavior. In some vertebrates, such as ruminants, gut microbes are so essential that the host evolved specialized organs to enhance gut microbial functionality. These observations indicate that the gut microbiome may play a significant role in the evolution of vertebrates. But if so, how?

Recent efforts to answer this question center on linking the taxonomic diversity of the gut microbiome to vertebrate evolution. Several studies found that the difference in gut microbiome biodiversity among vertebrates correlates with their evolutionary history (1–4). Related research observed signatures of microbial heritability (reviewed in reference 5) and patterns of cophylogeny between specific microbes and their hosts (2, 3, 6). While these associations are consistent with the hypothesis that the gut microbiome influences vertebrate evolution, potential confounding factors, such as differences in host diet or biogeography (1–3, 7), complicate their interpretation. Moreover, a variety of processes could produce these associations (4). For example, vertebrates evolved diverse gastrointestinal traits that may select for specific microbial assemblages and produce host species-specific microbiome signatures. Under such a situation, the microbiome could have no effect on vertebrate evolution or this could be a mechanism by which vertebrates select for essential taxa.

By additionally considering the myriad functions that the microbiome executes, we can better clarify how it contributes to phenotype and how significantly its contribution matters to fitness. For example, does the gut microbiome’s production of essential vitamins relax selective pressure on the host to obtain these vitamins through the diet? Perhaps the acquisition of gut microbes that can degrade dietary toxicants or improve otherwise low-quality energy sources (such as woody feedstock) enables niche expansion of the host population and ultimately speciation. While assessment of microbial taxonomy may reveal indicators of microbiome-mediated selection, assessment of microbial function provides a direct inroad into the mechanisms underlying any such selection. Additionally, many different microbial species may execute functions that are effectively redundant in terms of their contribution to phenotype. As a result, strong relationships may appear to exist between host physiology and microbiome function that are masked through only the consideration of microbial taxonomy. This functional redundancy may explain how vertebrates can experience different environments or stochastic ecological processes (e.g., microbial dispersal), both of which have the potential effect of exposing the gut to different microbial community assemblages, without a loss in fitness.

Recent innovations enable us to investigate the relationship between microbiome function and vertebrate evolution. Metagenomic functional annotation provides direct insight into the kinds of genes that are carried in the gut microbiome and consequently can profile the microbiome’s functional capacity (8). Advances in metabolomic methodology provide quantitative insight into microbiome chemotype. Emerging statistical applications can reveal how microbiome genes or metabolites associate with host health (9). Experimental tools, such as high-throughput culturing, gnotobiotic organisms, and microbiome transplantations, provide empirical readouts on the functional effect of microbiomes or specific microbes on host phenotype. While future developments will improve the precision of these technologies, they currently offer an opportunity to transform how we assess the microbiome’s contribution to fitness.

Cutting-edge research demonstrates the potential for these resources to clarify how the microbiome influences vertebrate evolution. In a recent study, microbiomes were transplanted among rodents to reveal phylogenetically optimized effects of the microbiome on feed efficiency (4). This finding ties the functional effect of the microbiome to the evolution of these rodents. Similarly, lab mice improved infection resiliency if they received transplants of wild mouse stool (10), indicating that artificial environments may facilitate microbiome drift due to relaxed selection. A culture-based investigation identified oxalate-degrading gut bacteria that promoted a rodent’s dietary niche expansion (11), suggesting that the microbiome contributed to adaptation. A gnotobiotic zebrafish study found that transplanted human gut bacteria encode functions that are redundant with zebrafish gut microbiota and that are critical to beta-cell expansion (12). These conserved functions may be subject to purifying selection.

We should capitalize upon these recent innovations and discoveries by answering foundational questions about the relationship between microbiome function and vertebrate evolution.

## HOW DOES GUT MICROBIOME FUNCTION VARY AMONG VERTEBRATE SPECIES?

**FIG 1:**
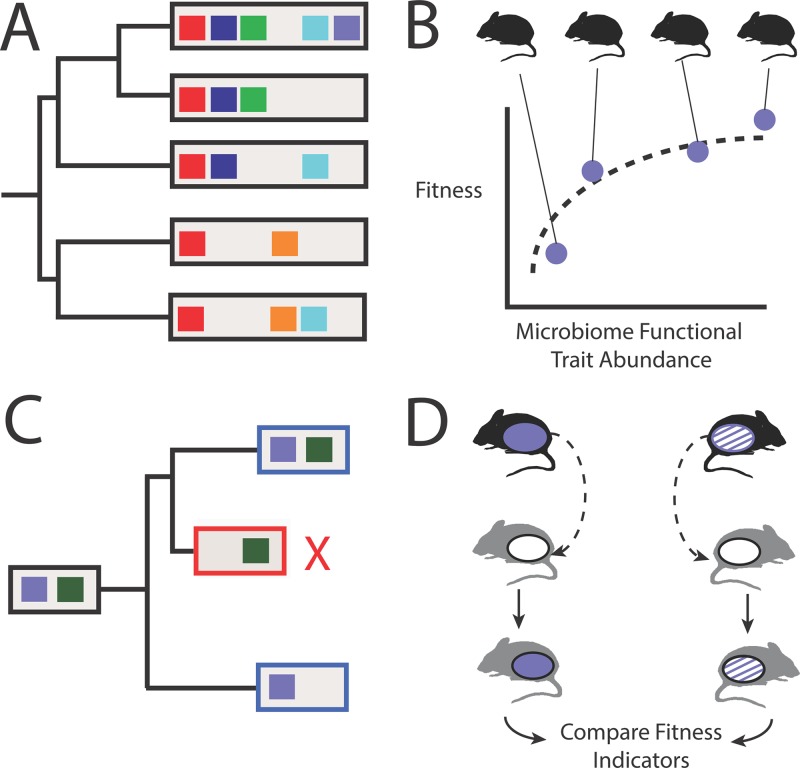
Conceptual illustrations of how microbiome function can be studied as an evolutionary trait. (A) Quantification of how gut microbiome functions (colored boxes) distribute across vertebrate species can reveal which functions manifest phylogenetic signal and are potential vertebrate evolutionary traits. (B) Population-level analyses of microbiome function can demonstrate how specific functions vary among individuals and associate with fitness. Microbiome functions that correlate with fitness can be further investigated to validate their contribution to host fitness and discover the demographic processes that impact their population-level distribution. (C) Observations of microbiome functions in extant lineages (boxed in blue) and neutral models of how microbiomes change throughout host evolution can theoretically predict ancestral microbiome functions (boxed in black). Moreover, neutral models can consider the distribution of microbiome functions among extant lineages to quantify rates of microbiome functional conservation and diversification and identify specific functions that putatively affect host fitness (e.g., deleterious effects from loss of conserved functions [boxed in red]). (D) Ultimately, studies must validate microbiome function fitness effects. Validation can come in many forms, including transplantation of microbiomes that encoded a fitness-associated function (purple ellipse) from wild individuals (black mice) into gnotobiotic animals (gray mice). Comparison of the physiological effects of transplantation across a donor population that varies in the abundance of the microbiome function (hashed purple ellipse) can provide additional insight.

Comparative genomics revealed rates and patterns of genetic evolution that clarified the genomic basis of fitness. We should similarly seek to characterize how microbiome function varies across the vertebrate tree of life and define evolutionary patterns and processes of microbiome functional diversification (Fig. 1A). We know little about which functions the gut microbiome encodes outside of a small number of vertebrate species. Additionally, most of what we do know comes from facility-managed animals, which may not reflect the microbiomes found in those living in natural habitats. Few studies have compared the microbiome’s functions across vertebrate species, but those that have offer evidence of both functional conservation (7, 13) and lineage-specific divergence (14).

By expanding our knowledge of microbiome function across vertebrates, especially from wild populations, we can clarify how microbiome function has diversified as vertebrates evolved. For example, some functions may be conserved across distantly related host species, potentially because they are integral to host fitness. Others may be unique to specific host lineages, thereby indicating that they may have played a role in adaptive evolution, especially if closely related species are being compared. Of course, correlation does not prove causation; these associations could arise for reasons other than selection acting on the host. Still, quantification of the distribution of microbiome functions across vertebrates can reveal important candidate functions involved in vertebrate evolution and provide foundational insight into the evolutionary rates of microbiome functional diversification.

## WHAT IS THE RELATIONSHIP BETWEEN POPULATION DEMOGRAPHICS AND GUT MICROBIOME FUNCTION?

While interspecies comparisons can illuminate macroevolutionary trends, insight into specific evolutionary mechanisms may be best gleaned through population-level studies of microbiome function (Fig. 1B). For example, integration of host genotype and microbiome data can reveal how demographic processes, such as migration, bottlenecks, and inbreeding, relate to the interindividual variation in microbiome function. Moreover, these analyses can provide insight into the heritability of microbiome function, which may be more heritable than microbiome taxonomy due to functional redundancy.

Population-level analyses can also provide insight into the relationship between microbiome function and vertebrate natural selection. Perhaps the populations that harbor specific microbiome functions or increased functional diversity are those that better resist or respond to ecological perturbations. Genome-wide association studies can reveal polymorphisms that associate with specific features of the gut microbiome. Such polymorphisms may represent host genomic mutations that are subject to selection because they recruit specific functional assemblages in the microbiome. Moreover, by pairing the collection of microbiome data with measures of ecophysiology, we can discern if the microbiome stratifies individuals in wild populations based upon these proxy measures of ecological fitness.

## TO WHAT EXTENT DO NEUTRAL PROCESSES INFLUENCE GUT MICROBIOME FUNCTION?

Selection can only be invoked as an evolutionary mechanism if the characteristics in extant individuals are unlikely to have arisen due to chance. Indeed, natural selection is not required to evolve traits. Random processes, such as genetic drift and genetic draft in the case of genomic evolution, can yield trait distinctions between vertebrate species. Such traits are less relevant to our understanding of vertebrate adaptation and must be distinguished from those that evolved in response to natural selection. Neutral models are critical in this regard as they provide an expectation of how traits distribute across populations and species given random ecological and evolutionary processes (Fig. 1C). Traits subject to natural selection are those that significantly diverge from the neutral expectation.

Neutral models of host-microbiome coevolution are emerging (5), but they tend to focus on microbial taxonomy. We need neutral models that predict how microbiome function diversifies so that we can discern how natural selection acts on it to influence vertebrate evolution. Ideally, these models will consider the evolutionary ecology of gut microbes, including their dispersal between individuals, heritability across host generations, and colonization and successional dynamics in the gut. Moreover, these models should account for how the environmental community of microbes to which an individual is exposed (e.g., foodborne microbes) influences the gut microbiome. Models should also consider that evolution may select for host genomic variants that in turn select for specific functional assemblages in the microbiome and that the specificity of the host’s selection on the microbiome may be varied. By incorporating these and related parameters into a probabilistic framework, we ultimately can identify specific observations of microbiome function that differ from the expectations of the model, such as those that are more uniform or variable across vertebrates than expected by chance. We will also need methods to distinguish selected microbiome functions from those that hitchhike due to linkage. While the evolutionary predictions produced by these models will require validation (and refinement), their development can contribute to new theoretical frameworks in evolutionary biology that expedite the discovery of microbiome determinants of vertebrate evolution.

## DOES MICROBIOME FUNCTION MATTER TO FITNESS?

While associative studies can clarify how gut microbiome function relates to vertebrate evolution, we will need to validate their contribution to vertebrate fitness. Interspecies microbiome transplantation and monoassociation studies can reveal the physiological effects of swapping microbiomes across species and colonizing the gut with specific microbes, respectively (Fig. 1D). However, these approaches are limited to considerations of bulk community diversity or currently cultured microbes, which hinders the discovery of specific microbiome functional pathways that impact physiology and fitness. Efforts to expand the vertebrate gut microbiome culture collection can provide added opportunities to test specific hypotheses. Likewise, we can adapt synthetic biology techniques to knock down specific organisms from a community, and consequently their functions, or knock-in genetic pathways into well-studied gut strains to measure the pathway’s effect on phenotype. Furthermore, functional products, such as specific metabolites, can be synthesized and fed to lab-managed animals to measure their physiological effects. Expanding assessments of physiology to include well-studied indicators of fitness, such as fecundity, can also improve hypothesis testing.

## CONCLUSION

Robust answers to these foundational questions are prerequisite to understanding how the gut microbiome contributes to vertebrate evolution. While answering these questions will be challenging, it is worth the effort given the social (i.e., economic, environmental, and human health) value of this knowledge. This research holds great potential for revealing microbiome products that influence physiology and can consequently serve as therapeutic leads. It can resolve microbiomic indicators of wildlife population health that can help conservation biologists predict and manage population declines. It can explain the physiological consequences of the disappearing microbiome hypothesis (15) and clarify how ancestrally evolved gut microbiome functions can be restored through supplementation with functionally redundant taxa. Ultimately, this research will illuminate the role of the microbial biosphere in the origin of our species to better explain how we came to be.
